# Selection of Parental Material to Maximize Heterosis Using SNP and SilicoDarT Markers in Maize

**DOI:** 10.3390/plants8090349

**Published:** 2019-09-14

**Authors:** Agnieszka Tomkowiak, Jan Bocianowski, Dominika Radzikowska, Przemysław Łukasz Kowalczewski

**Affiliations:** 1 Department of Genetics and Plant Breeding, Poznań University of Life Sciences, 11 Dojazd St, 60–632 Poznań, Poland; 2 Department of Mathematical and Statistical Methods, Poznań University of Life Sciences, 28 Wojska Polskiego St, 60–637 Poznań, Poland; jan.bocianowski@up.poznan.pl; 3 Department of Agronomy, Poznań University of Life Sciences, 11 Dojazd St, 60–632 Poznań, Poland; dominika.radzikowska@up.poznan.pl; 4 Institute of Food Technology of Plant Origin, Poznań University of Life Sciences, 31 Wojska Polskiego St, 60–624 Poznań, Poland; przemyslaw.kowalczewski@up.poznan.pl

**Keywords:** heterosis, molecular markers, SNP, SilicoDArT, *Zea mays* L.

## Abstract

The chief aim of plant breeding is to improve varieties so as to increase their yield and breeding traits. One of the first stages of breeding is the selection of parental forms from the available gene pool of existing varieties. To date, costly and laborious methods based on multiple crossbreeding and phenotypic selection have been necessary to properly assess genetic resources in terms of productivity, quality parameters, and susceptibility to biotic and abiotic stressors. The often long and complicated breeding cycle can be significantly shortened through selection using DNA markers. To this end, use is made of close couplings between the marker and the locus responsible for the inheritance of the functional trait. The aim of this study was to identify single nucleotide polymorphism (SNP) and SilicoDArT markers associated with yield traits and to predict the heterosis effect for yield traits in maize (*Zea mays* L.). The plant material used in the research consisted of 19 inbred maize lines derived from different starting materials, and 13 hybrids resulting from crossing them. A two-year field experiment with inbred lines and hybrids was established at two Polish breeding stations on 10 m^2^ plots in a randomized block design with three replicates. The biometric measurements included cob length, cob diameter, core length, core diameter, number of rows of grain, number of grains in a row, mass of grain from the cob, weight of one thousand grains, and yield. The isolated DNA was subjected to DArTseq genotyping. Association mapping was performed in this study using a method based on the mixed linear model with the population structure estimated by eigenanalysis (principal component analysis of all markers) and modeled by random effects. Narew, Popis, Kozak, M Glejt, and Grom were the hybrids used in the study that showed the highest significant heterosis effect in 2013 and 2014. The similarity between parental components determined on the basis of SNP and SilicoDArT marker analysis did not exceed 33%. It was found that the genetic similarity between parental components, determined on the basis of SNP and SilicoDArT markers, reflected their degree of relationship, and correlated significantly with the effect of heterosis. As the results indicate, the parental components for heterosis crosses can be selected based on genetic similarity between parental components evaluated using SNP and SilicoDArT markers, supported with information on the origin of parental forms. Of the markers we analyzed, 76 were selected as being significantly associated with at least six traits observed in 2013 and 2014 at both the Łagiewniki and Smolice stations.

## 1. Introduction

The pressure to increase and sustain food production has been felt for a long time. Tools have thus been developed to guarantee greater accuracy in selection. The currently used methods of selection have been enhanced by the achievements of molecular biology and statistical models, enabling identification of both the markers of individual traits resulting from the action of individual genes and those conditioned by many QTLs that explain the phenotypic traits to various extents [1].

The DArT marker can be used in genomic selection (GS) [2]. GS allows for plant selection based on the total pool of DNA markers for the selected statistical model. It reduces the need for phenotyping and shortens the culture cycle. Meuwissen first described this method; he examined the accuracy of genomic selection carried out using the DArT technique and compared this with phenotypic selection and with selection supported by molecular markers (marker assisted selection, MAS). Genomic selection proved to be 28% more accurate than traditional marker-assisted selection, though slightly less accurate than phenotypic selection. The results of his study demonstrate that GS can be used to increase the profitability of breeding [3]. The method has been successfully used in barley [4] and oats [5], and also works well in improving the efficiency of breeding perennial species, such as eucalyptus (Eucalyptus L’Her) [6].

Modern methods for identifying single nucleotide polymorphisms (SNPs) make use of next generation sequencing (NGS) methods. These refer to sequencing techniques developed in the twenty-first century that provide higher performance and throughput than the Sanger [7] sequencing technique commonly used before. The most common NGS techniques are pyrosequencing 454 [8], the Solex technique (Ilumina), the SOLiD platform (Applied Biosystems), the Polonator system (Dover/Harvard), and the HeliScope single molecule sequencer (Helicos). These technologies provide inexpensive whole-genome sequence readings through methods such as chromatin immunoprecipitation, mutation mapping, detection of polymorphisms, and detection of noncoding RNA sequences [9]. Modern sequencing methods enable the identification of a large number of markers and also allow more accurate examination of many loci.

Modern genotyping technologies can also shed new light on the genetic basis of heterosis. The use of heterosis to increase and stabilize yield has become one of the major drivers of increased agricultural production over the last few decades. Despite the huge significance of heterosis and the growing tendency to use hybrid vigor even in inbred crops like bread wheat, the molecular and genetic mechanisms underlying this phenomenon have still not been fully explained [10].

Song and Messing [11] isolated a specific region of the genome of two crossed inbred corn lines, which were subsequently sequenced and mapped. They found that the size of this area and the presence of genes from a given gene family in it were significantly different. Genes that were present in one line were absent in the other, although phenotypic symptoms of their expression were visible in the other line. This is evidence that genes from the same gene family that produce similar phenotypic effects were located in different parts of the genome in each of the tested lines. According to Song and Mesing, heterosis can therefore be a consequence of differences in the structure of the genome, especially in the distribution and presence of certain genes from a given gene family in crossed inbred lines. Predicting the magnitude of the heterosis effect in hybrids based on molecular marker analysis has been widely discussed. According to the literature, there is regression of either hybrid performance or heterosis with increasing molecular genetic distance and estimation of correlations between these variables [11,12,13,14] or estimation of marker effects and marker associations with hybrid performance, heterosis, or specific combining ability [12,15].

The aim of this study was to identify single nucleotide polymorphism (SNP) and SilicoDArT markers associated with yield traits and to predict the heterosis effect for yield traits in maize (*Zea mays* L.). This topic was selected because the decreasing cost of next-generation sequencing means that these methods are beginning to be used in applied research to identify feature markers or even to select on the whole-genome level. This publication is one of a number to recently have suggested the possibility of using the latest molecular techniques (such as SNP and SilicoDArT) to select parental materials for heterosis crosses.

## 2. Results

### 2.1. Phenotyping

Analysis of variance indicated that the main effects of year and genotype—as well as the L×G, Y×G, and L×Y×G interactions—were significant for all the studied traits. The main location effects were not significant for LCO or MGC. The L×Y interaction was not significant for NGR or MGC.

Table 1 and Table 2 show trait correlation matrices for both locations and years. All significant coefficients were positive. Most trait pairs were correlated in all four environments. Three pairs (LC-NRG, LCO-NRG, and NRG-WTG) were not significant in any of the four environments. Additionally, LC-DCO, LCO-DCO, DCO-NGR, DCO-MGC, DCO-WTG, DCO-Yield, NRG-NGR, NRG-MGC, and NRG-MGC were not significant at Łagiewniki 2012 (Table 3). NRG was not correlated with yield in either year at Łagiewniki.

**Figure 1:**
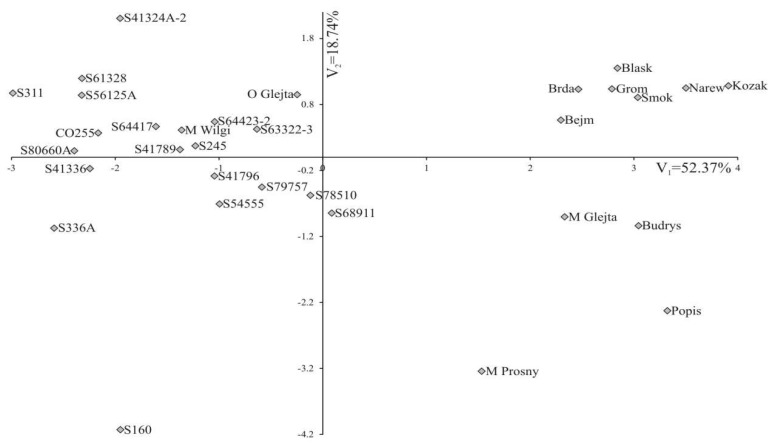
Distribution of maize (*Zea mays* L.) genotypes in the system of the first two canonical variables.

Individual traits are of differing importance and represent different proportions in the joint multivariate variation. Analysis of the multivariate genotypic variation also includes identification of the most important traits in the multivariate variation of genotypes. Analysis of canonical variables is a statistical tool that makes it possible to solve the problem of multivariate relationships. The first two canonical variables jointly explain 71.11% of the total variation between genotypes (Figure 1).

Figure 1 presents trait variation in the analyzed genotypes in the system of the first two canonical variables. In the graph, the point coordinates of a given genotype constitute the values of the first and second canonical variables. Three groups containing inbred lines and hybrid forms can be distinguished in Figure 1. The first group consists of the O Glejt hybrid and all the inbred lines that exhibited inbred depression for all analyzed yield structure parameters, except for the S41324A-2 and S160 lines. The second group consists of the hybrid forms, which have the same paternal components (Brda, Blask, and Grom), where the S41324A-2 line was the paternal form, and Bejm, Dragon, Narew, and Kozak, where the S61328 line was the paternal form. The third group consists of hybrids (M Glejt, M Prosny, Budrys, and Popis), whose parental components were not related to each other or else were related only to a small percentage. The first canonical variable was significantly positively correlated with LC, DC, LCO, NGR, MGC, WTG, and yield. The second canonical variable was significantly negatively correlated with NRG. The greatest variation in terms of all the traits considered together (measured using Mahalanobis distances) was found for S160 and Kozak (with a Mahalanobis distance of 8.01). The greatest similarity was found for the S64423-2 and M Wilga genotypes (0.91). The Mahalanobis distance values for all genotype pairs are presented in Table 4.

### 2.2. Genotyping Data

#### 2.2.1. SilicoDArT and SNP

**Figure 2:**
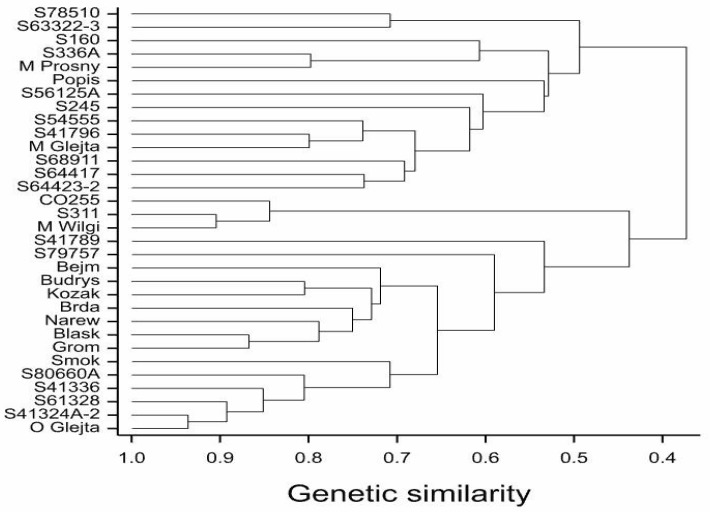
Dendrogram of genetic similarity of 19 inbred lines and 13 hybrids of maize (*Zea mays* L.) on the basis of 8192 marker observations.

DArTseq NGS analysis of the tested maize lines allowed us to identify 49,911 polymorphisms (33,452 SilicoDArTs and 16,459 SNPs). In total, 8192 of these markers (including 8189 SilicoDArTs and three SNPs) were selected for GWAM using the criteria specified above. The dendrogram (UPGMA) based on these 8192 markers showed the genetic relationships of 19 inbred lines and 13 hybrids (Figure 2).

The dendrogram shows three basic similarity groups. The first group includes the M Prosny hybrid with its parental line, the Popis hybrid with its maternal line, the M Glejt hybrid, and the inbred lines S78510, S63322-3, S56125A, S245, S41796, S68911, S64417, and S64423-2. The second group contains the M Wilga hybrid with its parental components. The third group consists of the hybrids Bejm, Budrys, Kozak, Brda, Narew, Blask, Grom, and Smok with their paternal lines and the O Glejt hybrid with its maternal line (Figure 2). The highest genetic similarity, calculated on the basis of both types of markers (equal to 0.94) was detected between S41324A-2 and O Glejt, whereas the lowest genetic similarity (equal 0.17) was found for S41796 and S61328. Figure 2 shows that the genetic similarity (as determined on the basis of the SNP and SilicoDArT markers) between the parental components of individual hybrids reflects their relative relationships, except in the case of the parental lines of the Blask hybrid (Table 1). The highest genetic similarity (83%, 80%, and 52%) was recorded between parental components of the O Glejt, M Wilga, and Grom hybrids, whose relationship between parental components was 50% (Table 3).

#### 2.2.2. Association Mapping

There were 1678 markers that were significantly associated with the investigated traits at FDR < 0.05 in GWAM: 1675 SilicoDArTs and three SNPs. Table 5 shows the number of markers relevant for individual traits in the considered environments. We observed a large number of statistically significant associations with particular features: 3003 (for LC), 3750 (for DC), 2901 (for LCO), 1641 (for DCO), 851 (for NRG), 2097 (for NGR), 3419 (for MGC), 3886 (for WTG), and 2128 (for Yield).

Seventy-six of the analyzed markers were selected as being significantly associated with at least six traits observed in 2013 and 2014 at both Łagiewniki and Smolice (Table S1). The most significant marker was 4777143, which determined all the analyzed traits except for NGR and DCO at Łagiewniki in 2013. The following markers were significantly associated with the yield in both localities in 2013 and 2014: 4777143, 100002778, 4767650, 21693206, 9625858, 16723979, 9713903, 100002999, 100000002, and 7057018. The markers most often significantly associated with the observed features are shown in Table S1.

#### 2.2.3. Prediction of the Heterosis Effect

The values of heterosis effects for the observed traits in individual environments are shown in Table 6. Narew and Popis were the hybrids that showed the highest significant heterosis effect for most of the analyzed yield structure traits at Łagiewniki in both 2013 and 2014 (Table 6). The Narew hybrid showed the most significant heterosis effect for DC (0.778), MGC (89.9) and WTG traits (136.4) in 2014, and for LC (5.99), LCO (5.32), MGC (111.8), and WTG traits (146.8) in 2013. The Popis hybrid showed the greatest heterosis effect for NRG (2.03), NRG (11.07), and Yield (7.72) in 2013, and for NRG (1.93) and Yield (9.79) in 2014. The highest significant heterosis effects for the three examined traits were also recorded in the Kozak hybrid in 2013 and for four analyzed traits in the M Glejt hybrid in 2014. It is noteworthy that the parental components of these four hybrids were either not related to each other or else showed a low degree of relationship due to origin (Narew: 4% relationship between parents; Popis: 0%; Kozak: 0%; and M Glejt: 13%). The similarity between parental components, as determined on the basis of SNP and SilicoDArT markers was also low (Narew: 18% similarity between parents; Popis: 26%; Kozak: 26%; and M Glejt: 33%; Table 3). The situation was similar for both years at Smolice. Here, Narew turned out to be the best hybrid, showing the most significant heterosis effect for the DC (0.897), NRG (2.37), and WTG traits (118.8) in 2013, and for the DC (0.847) and WTG traits (151) in 2014. The highest significant heterosis effects for two traits were also recorded in the Popis and Grom hybrids in 2013 and for four traits in the Kozak hybrid in 2014 (Table 6).

The relationships between the effects of heterosis and genetic and phenotypic distance (expressed as the Mahalanobis distance) are shown in Table 7. Statistical analysis shows the features of the yield structure, for which the magnitude of the heterosis effect in the hybrid forms depended on the genetic distance between the parental components estimated on the basis of SNP and SilicoDArT markers.

As shown in Table 7, the scale of the heterosis effect for most of the observed traits was significantly correlated with genetic distance, regardless of the year and locality of the experiment. MGC was the most significantly correlated (0.8689*** in 2013 at Łagiewniki, 0.8646*** in 2014 at Łagiewniki, 0.7835** in 2013 at Smolice, and 0.8834*** in 2014 at Smolice). The LC, LCO, and Yield traits were also highly significantly correlated. Regarding the phenotypic distance expressed by the Mahalanobis distances, no significant correlations were found with the magnitude of the heterosis effect in the hybrid forms, except for the four following features: Yield in 2013 at Łagiewniki and LC, LCO, and WTG in 2013 at Smolice.

## 3. Discussion

Maize is a major crop species characterized by very high yield efficiency and versatility in utilizing the whole plant. Modern breeding programs focus on hybrid cultivars with the greatest heterosis effect, thanks to which it is possible to obtain much higher yields through appropriate selection of parental components.

Heterosis or hybrid vigor is a phenotypic result of gene interaction due to the effect of heterozygotes of hybrids in the F1 generation. According to the dominance hypothesis cited by Ruebenbauer, having many heterozygous genes causes an increase in hybrid performance due to the dominant alleles [16]. The more homozygous alleles are complementary in the parental forms, the greater the effect of heterosis in the hybrid forms. Hence, the greatest theoretical heterosis can be expected when there is a large allele diversity of individual genes in the parent plants. Such diversity occurs when the crossed genotypes are less related and the genetic distance is greater. Progeny of genotypes with a large genetic distance should thus show a significant heterosis effect.

We used the SilicoDArT and SNP markers to assess genetic diversity. The DArTseq NGS analysis of the tested maize lines allowed us to identify 49,911 polymorphisms (33,452 SilicoDArT and 16,459 SNP). In total, 8192 of these markers (including 8189 SilicoDArT and three SNPs) were selected for GWAM. Of all the analyzed markers, 76 were selected as being significantly associated with at least six traits observed in 2013 and 2014 at both Łagiewniki and Smolice.

In the present study, the genetic distance between parental components, as determined by the SNP and SilicoDArT markers, reflected their degree of relationship and was significantly correlated with the heterosis effect observed in the majority of the yield structure features, as well as the yield itself. Genotypes grouped according to specific patterns are shown on the dendrogram, with the first group including all inbred lines, except for the S41324A-2, S160, and O Glejt hybrid lines. The second group consists of hybrid forms that have the same paternal components. The third group consists of hybrids (M Glejt, M Prosny, Budrys, and Popis) whose parental components were not related to each other, or which were only related to a small percentage.

As the results indicate, the parental components for heterosis crosses can be selected on the basis of genetic distance between the parental components, as determined using SNP and SilicoDArT markers, supported with information on the origin of the parental forms.

In this study, Narew, Popis, Kozak, M Glejt, and Grom were the hybrids that showed the highest significant heterosis effect for the majority of the yield structure traits, at both sites in both years. Importantly, the parental components of these hybrids (except for the Grom hybrid) were either not related to each other or else showed only a low degree of relationship due to origin (Narew: 4% relationship between parents; Popis: 0%; Kozak: 0%; and M Glejt: 13%). The similarity between parental components determined on the basis of SNP and SilicoDArT marker analysis did not exceed 33% (Narew: 18% similarity between parents; Popis: 26%; Kozak: 26%; and M Glejt: 33%). Many researchers attribute the dependence of the heterosis effect on the genetic distance of parental forms, taking into account their degree of relationship [17,18,19,20,21]. In our own research, the genetic distance between parental components, as estimated with SNP and SilicoDArT markers, reflected their relationship and translated into the magnitude of the heterosis effect. We observed that the lower the similarity and the degree of relationship between parental components, the greater the effect of heterosis was in the hybrid forms.

In recent years, methods have been sought to allow initial selection of lines intended for heterosis crossing. The dependence of the heterosis effect on genetic distance, as determined using molecular markers, has been analyzed by many researchers in various species, including [22], pepper [23], cocoa [24], barley or sunflower [25]. Factors associated with the hybrid heterosis effect resulting from the crossing of inbred maize lines were discovered in 1992 [26]. Research conducted using molecular RFLP markers on 148 inbred lines of maize supported the use of these markers when the breeding material was clustered into heterotic groups [27]. The AFLP system was used to select parental components for maize heterosis crosses [28]. Five primer pairs generated 56 polymorphic bands, allowing the degree of similarity to be determined, which was then correlated with the effect of heterosis. Shehata et al. [29] demonstrated the usefulness of the SSR system in assessing the genetic distance of eight inbred maize lines. Berilli et al. [30] studied the genetic distance between two maize populations (CYMMYT and Piranao), which was estimated using molecular ISSR markers. Thirteen primers generated as many as 140 products, of which 84.4% were polymorphic. The genotypes tested were divided into two main groups, which contained mainly individuals from a single population.

DArT technology also works as an efficient diagnostic tool for analyzing genetic diversity [31]. DArT markers have been successfully used to study the genetic diversity and structure of Chinese common wheat (*Triticulum aestivum* L). A total of 111 cultivars and breeding lines from northern China were examined, with the results providing information that allowed further selection of parental forms and the establishment of heterozygous materials for the needs of the Chinese wheat breeding program [32]. The DArT method has found broad application in relationship analysis, such as in oats (*Avena* sp.), where 134 cultivars were examined and groups corresponding to winter and spring forms were identified [33]. However, research into 232 forms of the pigeon pea (*Cajanus cajan*) showed a low degree of material differentiation. Of 696 DArT markers, only 64 turned out to be polymorphic, with the wild forms being the most diverse [34].

Genome profiling in large hybrid populations currently offers unprecedented resolution for the dissection of loci and genes involved in heterotic expression. Huang et al. [35] recently published a study in which an extensive population of 1495 elite hybrid rice varieties, along with their inbred parental lines, were subject to detailed genome-wide sequence analysis in order to investigate genomic effects on hybrid vigor for 38 agronomic traits. The resequenced genomes of all parental lines harbored around 1.3 million polymorphic SNP markers, which were subsequently used to study population genetic parameters and perform GWAS at an unprecedented resolution. This approach revealed heterozygous chromosome regions that contributed to trait expression in the F1 hybrids. Elucidation of the corresponding genomic effects on phenotypic traits demonstrated that the pyramiding of multiple loci facilitated the accumulation of many rare superior alleles with positive effects. In other words, dominance complementation contributes most to the heterosis effect in the hybrid rice production. A combination of forward and background selection using high-throughput genome screening tools [36,37] can thus significantly increase the breeding gain potential through the efficient exploitation of hybrid vigor.

The idea of genomic hybrid breeding, in which a genome-based prediction strategy using genomic sequence data is used to estimate the performance of the F1 progeny in hybrid breeding, was introduced in rice by Xu et al. [38]. These authors used over 250,000 SNP markers generated by resequencing 210 parental inbred lines from a training set of 278 randomly selected hybrids; this study demonstrated the power of marker-directed estimation of F1 hybrid yields in rice. The top one hundred predicted hybrids, from a total of 21,945 possible combinations between the parental accessions, were estimated to exceed the overall average yield by 16%. This means there was a significant improvement in the average selection gains, compared to conventional breeding and accelerated hybrid rice production.

## 4. Materials and Methods

### 4.1. Plant Material

The plant material used for the research consisted of 19 inbred maize lines derived from a range of starting materials, and 13 hybrids resulting from their crossing. Maize lines and hybrids came from Hodowla Roślin Smolice (Table 3).

### 4.2. Phenotyping

A two-year field experiment (2012, 2013) with inbred lines and hybrids was established on 10 m^2^ plots in a randomized block design in three replicates at two breeding stations owned by Plant Breeding Smolice, part of the Plant Breeding Acclimatization Institute Group, at Smolice (51°42′20.813′’N, 17°9′57.405′’E) and Łagiewniki (50°47′27′’N, 16°50′40′’E), Poland. One cob each was selected from ten plants of each replicate to perform biometric measurements on. Biometric measurements were carried out in the first half of November each year and included cob length (LC), cob diameter (DC), core length (LCO), core diameter (DCO), number of rows of grain (NRG), number of grains in a row (NGR), mass of grain from the cob (MGC), weight of one thousand grains (WTG), and Yield.

### 4.3. Genotyping and SilicoDArT and SNP Data Processing

The genotypic data for association mapping were derived from polymorphisms identified in DArT and candidate gene sequences.

### 4.4. DArT Sequences

Thirty-two genotypes were genotyped. The total genomic DNA extraction from the young leaves of the analyzed forms was performed using the GenElute Plant Mini Kit (Sigma-Aldrich, Darmstadt, Germany). DNA purity and concentration were determined spectrophotometrically (Thermo Scientific, Waltham, MA, USA), and the quality was determined electrophoretically in a 1% agarose gel. The concentration of all DNA samples was adjusted to 100 ng µl^–1^. DArTseq analysis was performed at Diversity Arrays Technology, Australia. The GBS procedure involves several stages, which include preparation of DNA samples, digestion of genomic DNA with restriction enzymes, ligation of adapters, and independent creation of individual libraries and their final assembly. Next, the products are amplified, and the results are sequenced and analyzed. The detailed methodology is as follows: DNA samples were processed in digestion/ligation reactions principally as per Kilian et al. [39], but replacing a single PstI-compatible adapter with two different adapters corresponding to two different restriction enzyme (RE) overhangs, and transferring the assay onto the sequencing platform, as described by Sansaloni et al. [40]. The PstI-compatible adapter was designed to include the Illumina flowcell attachment sequence, sequencing primer sequence, and a “staggered” barcode region of varying length, similar to the sequence reported by Elshire et al. [41]. The reverse adapter contained the flowcell attachment region and the NspI-compatible overhang sequence.

Only “mixed fragments” (PstI-NspI) were effectively amplified in 30 PCR cycles under the following reaction conditions: denaturation for 1 min at 94 °C, followed by 30 cycles of 20 sec at 94 °C, 30 sec at 58 °C, 45 sec at 72 °C, and final elongation for 7 min at 72 °C. After PCR, equimolar amounts of the amplification products from each sample of a 96-well microtiter plate were bulked and applied to c-Bot Illumina bridge PCR, before sequencing on an Illumina Hiseq2500. Single read sequencing was run for 77 cycles.

The sequences generated from each lane were processed using proprietary DArT analytical pipelines. In the primary pipeline, the fastq files were first processed to filter away poor quality sequences, applying more stringent selection criteria to the barcode region than to the rest of the sequence. In this way, assigning sequences to the specific samples carried in the “barcode split” step is very reliable. Approximately 2,500,000 (± 7%) sequences per barcode/sample were used in marker calling. Finally, identical sequences were collapsed into “fastqcall files”. These files were used in the secondary pipeline for DArT PL’s proprietary SNP and SilicoDArT calling algorithms (presence/absence of restriction fragments in representation; DArTsoft14). Only DArT sequences meeting the following criteria were selected for the association analysis: one SilicoDArT and SNP within a given sequence (69 nt), minor allele frequency (MAF) > 0.25, and < 10% missing observation fractions.

### 4.5. Statistical Analysis and Association Mapping

The Henderson method [42] was used to construct a relationship matrix using the full pedigree information. Firstly, the normality of trait distribution was tested using the Shapiro–Wilk normality test [43]. Relationships between the traits were estimated using correlation coefficients on the basis of means of genotypes for each location and year independently. The results were also examined using multivariate methods. The canonical variate analysis was applied in order to present a multitrait assessment of the similarity of the tested genotypes in a lower number of dimensions with the least possible loss of information [44]. This allows the genotype variation to be illustrated in a graphic form in terms of all observed traits. The Mahalanobis distance was suggested as a measure of “polytrait” genotype similarity [45], whose significance was verified by means of the critical D_α_ value referred to as the least significant distance [46]. The Mahalanobis distances were calculated for species. The coefficients of genetic similarity (S) of the investigated lines were calculated using the Nei and Li [47] formulas. The lines were grouped hierarchically using the unweighted pair group method of arithmetic means (UPGMA) based on the calculated coefficients. The relationship between lines was presented in the form of a dendrogram. Association mapping was performed using a method based on a mixed linear model with the population structure estimated by eigenanalysis (principal component analysis applied to all markers) and modeled by random effects [48,49]. All analyses were conducted in Genstat 18.2. The significance of associations between traits and SilicoDArT and SNP markers was assessed on the basis of *P*-values corrected for multiple testing using the Benjamini–Hochberg method [50].

### 4.6. Prediction of the Heterosis Effect

Heterosis effects for hybrids for each trait were estimated and tested by comparing a particular hybrid with the trait mean of both parents. Analysis was carried out using the GenStat 18 statistical package.

## 5. Conclusions

This study has demonstrated that molecular SNP and SilicoDArT markers may be useful in predicting hybrid formulas in maize, and could find application in selecting parental components for heterosis crossings. These markers can also be used in maize to group lines in terms of origin and lines with incomplete origin data. In the breeding programs, it proved possible to successfully use lines S54555 and S79757 for heterosis crosses; these were the parent components of the hybrid Popis; lines S64417 and S61326, which were the parental components of the Narew hybrid, also proved useful for heterosis crosses. Narew and Popis were the hybrids that showed the highest significant heterosis effect for most of the analyzed yield structure traits at Łagiewniki in both 2013 and 2014. It is worth noting that the parental components of these two hybrids were either not related to each other or else showed only a low degree of relationship due to origin (Narew: 4% relationship between parents; Popis: 0%). The similarity between the parental components, as determined on the basis of SNP and SilicoDArT markers was also low (Narew: 18% similarity between parents; Popis: 26%).

## Figures and Tables

**Table 1:** Correlation matrix for the traits studied at Łagiewniki in 2011 (above diagonal) and 2012 (below diagonal).

Trait	LC	DC	LCO	DCO	NRG	NGR	MGC	WTG	Yield
LC	1	0.642***	0.999***	0.102	0.038	0.917***	0.914***	0.791***	0.924***
DC	0.689***	1	0.626***	0.456**	0.577***	0.662***	0.852***	0.711***	0.747***
LCO	0.991***	0.628***	1	0.102	0.01	0.906***	0.901***	0.793***	0.909***
DCO	0.483**	0.729***	0.481**	1	0.299	0.073	0.206	0.204	0.093
NRG	0.191	0.575***	0.125	0.362*	1	0.208	0.326	−0.051	0.277
NGR	0.921***	0.691***	0.910***	0.491**	0.398*	1	0.907***	0.619***	0.895***
MGC	0.915***	0.883***	0.872***	0.583***	0.466**	0.921***	1	0.803***	0.945***
WTG	0.662***	0.757***	0.616***	0.424*	0.036	0.467**	0.722***	1	0.746***
Yield	0.847***	0.790***	0.801***	0.465**	0.323	0.812***	0.918***	0.723***	1

* *P* < 0.05; ** *P* < 0.01; *** *P* < 0.001.

**Table 2:** Correlation matrix for the traits studied at Smolice in 2011 (above diagonal) and 2012 (below diagonal).

Trait	LC	DC	LCO	DCO	NRG	NGR	MGC	WTG	Yield
LC	1	0.765***	0.999***	0.485**	0.281	0.935***	0.939***	0.814***	0.857***
DC	0.653***	1	0.762***	0.632***	0.595***	0.749***	0.888***	0.787***	0.732***
LCO	0.998***	0.629***	1	0.494**	0.262	0.931***	0.934***	0.817***	0.855***
DCO	0.597***	0.715***	0.591***	1	0.387*	0.369*	0.481**	0.468**	0.464**
NRG	0.201	0.675***	0.167	0.368*	1	0.369*	0.462**	0.158	0.445*
NGR	0.952***	0.683***	0.944***	0.548**	0.372*	1	0.926***	0.666***	0.777***
MGC	0.917***	0.872***	0.901***	0.633***	0.482**	0.927***	1	0.843***	0.866***
WTG	0.579***	0.739***	0.565***	0.473**	0.117	0.452**	0.702***	1	0.724***
Yield	0.831***	0.811***	0.815***	0.513**	0.455**	0.825***	0.929***	0.709***	1

* *P* < 0.05; ** *P* < 0.01; *** *P* < 0.001.

**Table 3:** Hybrid F1 parental lines and their relationship.

No.	Maternal Lines	Paternal Lines	Relationship of Parental Forms (%)	Genetic Similarity (SNP and SilicoDarT) of Parental Forms (%)	Hybrids F_1_
1	S160	S336A	3	45	M Prosna
2	S41336	S41324A-2	50	83	O Glejt
3	S78510	S80660A	6	30	Budrys
4	S54555	S79757	0	26	Popis
5	S245	S41789	13	33	M Glejt
6	S311	Co255	50	80	M Wilga
7	S64417	S61328	4	18	Narew
8	S41796	S41324A-2	50	18	Blask
9	S41789	S41324A-2	50	52	Grom
10	S56125A	S41324A-2	0	21	Brda
11	S63322-3	S61328	0	26	Kozak
12	S64423-2	S61328	8	19	Bejm
13	S68911	S61328	5	19	Smok

**Table 4:** Mahalanobis distances between maize (*Zea mays* L.) genotypes.

Genotype		1	2	3	4	5	6	7	8	9	10	11	12	13	14	15	16
S160	1	0															
S41336	2	4.36	0														
S78510	3	4.5	2.96	0													
S54555	4	5.25	3.47	4.03	0												
S245	5	5.1	3.4	2.48	4.35	0											
S311	6	5.48	2.12	4.12	3.45	3.44	0										
S64417	7	5.32	2.29	2.56	3.5	1.94	2.58	0									
S41796	8	5.58	3.98	4.07	2.76	3.1	3.63	3.06	0								
S41789	9	5.26	3.17	3.14	3.76	1.74	3.19	1.96	1.89	0							
S56125A	10	5.74	3.16	3.42	5.41	2.34	3.29	2.18	4.76	3.2	0						
S63322-3	11	5.22	3.19	1.4	4.56	2.06	3.81	2.55	4.27	3.1	2.9	0					
S64423-2	12	5	2.27	1.95	3.2	2.2	2.47	1.86	3.4	2.7	2.93	1.67	0				
S68911	13	4.85	3.59	1.74	3.8	2.23	4.47	2.47	3.02	2.28	3.83	2.5	2.72	0			
S336A	14	3.5	1.87	3.19	3.07	3.45	2.37	2.9	3.95	3.6	3.74	3.41	2.39	3.84	0		
S41324A-2	15	6.58	3.36	4.27	4.78	4.64	2.96	4.1	5.6	5.02	4.34	3.63	2.82	5.42	3.64	0	
S80660A	16	4.3	1.39	3.22	3.75	3.06	1.61	2.6	3.93	3.13	2.94	3.08	2.07	3.92	1.76	2.94	0
S79757	17	4.47	3.31	1.42	4.44	1.39	3.95	2.42	3.83	2.66	2.74	1.37	2.05	1.93	3.27	4.51	3.1
CO255	18	4.55	2.29	2.91	4.36	2.47	2.28	2.7	4.17	3.11	2.55	2.33	1.85	3.76	2.31	2.87	1.26
S61328	19	5.56	2.19	4.04	3.85	3.96	1.58	3.27	4.28	3.78	3.85	3.7	2.47	4.74	2.75	2.12	1.62
M Prosny	20	3.89	5.21	3.85	4.37	5.06	6.23	5.33	4.8	5.09	6.51	4.93	4.7	3.75	4.77	6.8	5.3
O Glejta	21	5.54	2.61	2.02	3.77	3.03	3.26	2.68	4.06	3.3	3.58	1.89	1.42	2.99	3.32	2.77	2.66
Budrys	22	6.03	5.65	3.45	5.75	5.26	6.77	5.52	6.15	5.78	6.24	4.26	4.6	4.08	5.85	6.18	5.77
Popis	23	5.67	6.1	4.25	5.84	5.96	7.31	6.12	6.35	6.25	7.01	5.29	5.38	4.58	6.16	7.13	6.28
M Glejta	24	5.8	4.87	3.19	3.94	4.52	5.74	4.4	4.33	4.48	5.82	4.16	3.88	2.98	5.09	5.96	5.15
M Wilgi	25	4.65	2.08	2.27	3.56	2.25	2.23	2.19	3.64	2.8	2.74	1.94	0.91	3.11	2.2	2.72	1.42
Narew	26	7.84	6.24	4.78	5.66	5.29	6.64	5.41	5.19	5.18	6.45	5.17	5.02	4.42	6.84	6.68	6.26
Blask	27	7.62	5.75	4.61	4.88	5.06	5.91	5.05	4.68	4.88	6.24	4.9	4.44	4.39	6.25	5.87	5.69
Grom	28	7.23	5.58	4.06	4.82	4.69	5.83	4.85	4.7	4.78	5.96	4.33	4.05	4.01	5.93	5.59	5.49
Brda	29	6.92	5.05	3.26	5.08	4.59	5.71	4.61	5.31	4.94	5.5	3.53	3.62	3.82	5.56	4.89	5.11
Kozak	30	8.01	6.65	4.44	6.8	5.67	7.38	6.01	6.76	6.24	6.54	4.68	5.2	4.87	7.14	6.47	6.67
Bejm	31	6.46	4.99	3.65	4.48	4.61	5.37	4.68	4.83	4.83	5.67	4.01	3.59	4.03	5.23	4.92	4.83
Smok	32	7.14	5.62	3.88	5.53	4.76	6.17	5.04	5.34	5.02	5.82	4.18	4.26	4.04	6.14	5.74	5.56
		**1**	**2**	**3**	**4**	**5**	**6**	**7**	**8**	**9**	**10**	**11**	**12**	**13**	**14**	**15**	**16**
**Genotype**		**17**	**18**	**19**	**20**	**21**	**22**	**23**	**24**	**25**	**26**	**27**	**28**	**29**	**30**	**31**	**32**
S79757	17	0															
CO255	18	2.45	0														
S61328	19	4.12	2.22	0													
M Prosny	20	4.25	5.42	6.04	0												
O Glejta	21	2.62	2.53	2.77	4.81	0											
Budrys	22	4.17	5.55	6.24	3.41	3.99	0										
Popis	23	4.91	6.26	6.83	2.57	4.99	1.67	0									
M Glejta	24	3.9	5.24	5.53	2.77	3.56	2.41	2.55	0								
M Wilgi	25	2.1	1.11	2.16	4.79	1.68	4.79	5.53	4.27	0							
Narew	26	5.06	6.19	6.3	4.98	4.44	3.61	4.13	2.72	5.3	0						
Blask	27	4.92	5.68	5.52	4.91	3.91	3.86	4.41	2.69	4.75	1.24	0					
Grom	28	4.38	5.33	5.42	4.48	3.56	3.18	3.9	2.32	4.4	1.49	0.94	0				
Brda	29	3.93	4.87	5.11	4.59	2.76	2.34	3.59	2.49	3.98	2.56	2.37	1.73	0			
Kozak	30	4.92	6.26	6.84	5.46	4.39	2.43	3.79	3.51	5.49	2.88	3.35	2.73	1.92	0		
Bejm	31	4.06	4.73	4.8	3.95	3.08	2.66	3.35	2.21	3.81	2.41	1.82	1.27	1.57	2.92	0	
Smok	32	4.23	5.32	5.66	4.56	3.53	2.49	3.47	2.51	4.48	1.56	1.8	1.28	1.33	1.8	1.57	0
		**17**	**18**	**19**	**20**	**21**	**22**	**23**	**24**	**25**	**26**	**27**	**28**	**29**	**30**	**31**	**32**

**Table 5:** The number of markers relevant to individual traits in relation to the environments.

Trait	Location	Year	Number of Significant Markers
LC	Łagiewniki	2013	679
		2014	865
	Smolice	2013	664
		2014	795
DC	Łagiewniki	2013	981
		2014	966
	Smolice	2013	979
		2014	824
LCO	Łagiewniki	2013	684
		2014	811
	Smolice	2013	695
		2014	711
DCO	Łagiewniki	2013	264
		2014	536
	Smolice	2013	300
		2014	541
NRG	Łagiewniki	2013	224
		2014	190
	Smolice	2013	227
		2014	210
NGR	Łagiewniki	2013	423
		2014	487
	Smolice	2013	553
		2014	634
MGC	Łagiewniki	2013	792
		2014	825
	Smolice	2013	876
		2014	926
WTG	Łagiewniki	2013	961
		2014	1045
	Smolice	2013	874
		2014	1006
Yield	Łagiewniki	2013	680
		2014	743
	Smolice	2013	102
		2014	603

**Table 6:** Heterosis coefficients for individual quantitative traits from the experiment carried out at Łagiewniki and Smolice in 2013 and 2014.

Hybrid	LC	DC	LCO	DCO	NRG	NGR	MGC	WTG	Yield
**Łagiewniki, 2013**									
Bejm	4.11***	0.653***	4.18***	0.042	0.93***	7.08***	77.9***	79.2***	4.57***
Blaks	4.09***	0.64***	3.89***	0.068*	0.97***	4.55***	70.5***	97.7***	7.72***
Brda	5.32***	0.597***	4.95***	0.107***	0.77**	10.47***	74.4***	85.9***	6.41***
Budrys	4.19***	0.523***	4.04***	-0.012	1.47***	7.52***	64.1***	68.6***	6.04***
Grom	5.18***	0.688***	5.19***	0.073*	1.5***	5.45***	75.9***	110.3***	6.24***
Kozak	5.63***	0.452***	5.65***	−0.217***	−0.77**	6.5***	76.6***	111.1***	5.85***
M Glejt	4.09***	0.498***	4.11***	0.137***	0.97***	8.13***	66.9***	29.4***	4.87***
M Prosna	3.54***	0.615***	3.34***	0.092**	1.43***	7.25***	65***	73.9***	4.58***
M Wilga	1.85***	0.038	1.88***	−0.087**	−0.13	4.02***	18.7***	19.5***	0.65
Narew	5.09***	0.778***	5.1***	0.068*	0.27	8.23***	89.9***	136.4***	4.97***
O Glejt	2***	0.238***	2.11***	−0.105***	−0.1	4.17***	33.9***	42***	1.21***
Popis	4.22***	0.378***	3.68***	−0.033	2.03***	11.07***	84.3***	12.1*	7.72***
Smok	4.6***	0.4***	4.72***	0	−0.37	7.13***	71.3***	95.2***	5.12***
**Łagiewniki, 2014**									
Bejm	5.73***	0.585***	4.96***	0.183***	1.8***	9.75***	82.1***	71.8***	4.97***
Blaks	4.66***	0.608***	3.52***	0.14***	-0.18	8.92***	93***	130***	5.92***
Brda	4.25***	0.862***	3.23***	0.207***	1.85***	12.8***	107***	123.8***	6.35***
Budrys	4.39***	0.382***	3.66***	0.015	1.07***	12.13***	81.2***	40.1***	8.49***
Grom	4.44***	0.845***	3.27***	0.233***	1.52***	8.5***	87.4***	123.1***	6.47***
Kozak	5.84***	0.395***	5.12***	−0.02	-0.2	12.9***	88.3***	99.4***	7.71***
M Glejt	5.74***	0.862***	4.78***	0.283***	1.93***	16.55***	107.5***	41.5***	6.87***
M Prosna	3.22***	0.73***	2.05***	0.115***	1.37***	5.02***	70.7***	115.8***	6.1***
M Wilga	1.43***	0.142***	1.11***	−0.018	0.2	4.47***	26.1***	33.6***	0.53
Narew	5.99***	0.735***	5.32***	0.182***	0.8**	11.93***	111.8***	146.8***	7.15***
O Glejt	1.46***	0.107**	1.3***	−0.002	-0.28	3.15***	20.5***	61.6***	0.26
Popis	4.23***	0.465***	2.84***	0.077**	1.93***	12.73***	96.1***	51.5***	9.79***
Smok	4.74***	0.357***	4.25***	0.11***	1.07***	9.15***	76.5***	72.6***	6.12***
**Smolice, 2013**									
Bejm	2.82***	0.597***	2.58***	0.092**	1.97***	3.73***	63.1***	59.3***	5.76***
Blaks	4.97***	0.747***	4.85***	0.19***	1.13***	8.22***	89.5***	110.6***	7.13***
Brda	5.09***	0.757***	5.08***	0.117***	1.33***	9.88***	70.6***	87.3***	6.03***
Budrys	4.92***	0.632***	4.82***	0.05	1.67***	10.23***	83.4***	64.2***	5.45***
Grom	5.54***	0.805***	5.32***	0.192***	1.23***	9.05***	96.9***	100.8***	6.05***
Kozak	5.32***	0.533***	5.53***	−0.078*	0.43	10.37***	85.7***	78.1***	5.37***
M Glejt	3.52***	0.622***	3.52***	0.19***	1.23***	11.35***	67.4***	13*	6.03***
M Prosna	4.18***	0.815***	3.77***	0.17***	1.7***	7.98***	77.2***	96.6***	5.96***
M Wilga	0.57	-0.008	0.72*	-0.122***	0.23	-0.7	2.4	-3.5	5.66***
Narew	5.43***	0.897***	5.39***	0.152***	2.37***	9.47***	109.5***	118.8***	7.05***
O Glejt	1.73***	0.14***	1.74***	-0.1**	0.07	1.97*	20.1***	44.4***	1.97***
Popis	5.27***	0.42***	4.95***	0.005	2.3***	13.63***	97.7***	41.1***	5.66***
Smok	4.96***	0.565***	4.87***	0.165***	0.6*	7.17***	80.8***	98.6***	4.6***
**Smolice, 2014**									
Bejm	4.54***	0.632***	4.58***	0.187***	1.23***	9.77***	89***	87.1***	4.59***
Blaks	4.14***	0.638***	4.26***	0.28***	0.47	9.82***	99.7***	93.5***	6.56***
Brda	4.78***	0.832***	4.68***	0.182***	1.77***	14.22***	106.1***	77.2***	7.64***
Budrys	5.61***	0.493***	5.56***	0.027	0.93**	13.13***	95.7***	44.9***	8.42***
Grom	3.94***	0.72***	3.7***	0.208***	1.53***	9.18***	90.4***	58.3***	6.7***
Kozak	6.17***	0.585***	6.27***	0.06*	0.17	14.25***	112.3***	98.9***	8.18***
M Glejt	4.03***	0.86***	3.92***	0.187***	2.27***	13.23***	98.8***	14.8**	6.25***
M Prosna	3.73***	0.717***	3.73***	0.183***	2***	9.75***	81.4***	65.9***	8.82***
M Wilga	1.31***	0.233***	1.35***	0.05	0.53	3.45***	27.5***	3.3	0.79
Narew	4.58***	0.847***	4.48***	0.223***	1.03***	8***	101.5***	151***	7.36***
O Glejt	0.91***	0.275***	1.02***	0.012	0.5	2.65***	29***	46.7***	0.23
Popis	3.64***	0.412***	3.55***	0.033	1.2***	10.32***	80.4***	38.8***	9.92***
Smok	5.55***	0.458***	5.67***	0.117***	0.53	9.8***	86***	72.9***	6.51***

* *P* < 0.05; ** *P* < 0.01; *** *P* < 0.001.

**Table 7:** Correlation coefficients between heterosis effects and genetic distance (calculated as 1-genetic similarity) and the Mahalanobis distance.

Environment	Trait	Heterosis vs. Genetic Distance	Heterosis vs. Mahalanobis Distance
Łagiewniki 2013	LC	0.786**	0.4009
	DC	0.6722*	0.2686
	LCO	0.7474**	0.3346
	DCO	0.3442	0.0412
	NRG	0.1855	0.1921
	NGR	0.6097*	0.0694
	MGC	0.8689***	0.3853
	WTG	0.4875	0.3798
	Yield	0.7974**	0.636*
Łagiewniki 2014	LC	0.866***	0.0541
	DC	0.5128	0.1244
	LCO	0.8038***	-0.0569
	DCO	0.4289	0.0368
	NRG	0.3757	-0.101
	NGR	0.7196**	-0.0635
	MGC	0.8646***	0.2388
	WTG	0.3698	0.521
	Yield	0.7836**	0.3294
Smolice 2013	LC	0.7396**	0.6372*
	DC	0.7034**	0.3322
	LCO	0.7345**	0.6191*
	DCO	0.5678*	0.2921
	NRG	0.5939*	0.0465
	NGR	0.6669*	0.3543
	MGC	0.7835**	0.5142
	WTG	0.5307	0.6541*
	Yield	0.5831*	0.1302
Smolice 2014	LC	0.8557***	0.2423
	DC	0.5874*	0.0114
	LCO	0.8536***	0.2437
	DCO	0.4611	0.2238
	NRG	0.1259	-0.2422
	NGR	0.7497**	0.1312
	MGC	0.8834***	0.2768
	WTG	0.5938*	0.3201
	Yield	0.7408**	0.4018

* *P* <0.05; ** *P* < 0.01; *** *P* < 0.001.

## Supplementary Materials

The following are available online at https://www.mdpi.com/2223-7747/8/9/349/s1, Table S1: The list of markers most often significantly associated with the observed features.
