# Supplementary material for: Selection of Parental Material to Maximize Heterosis Using SNP and SilicoDarT Markers in Maize
Source: Plants (Basel). 2019 Sep 14;8(9):349. doi: 10.3390/plants8090349 (PMC6783910; doi:10.3390/plants8090349)
Supplement: Supplementary file 1 [file plants-08-00349-s001.pdf]

Supplementary Material

Table S1. The list of markers most often significantly associated with the observed features.

Marker name	LC				DC				LCO				DCO				NRG				NGR				MGC				WTG				Yield			
	L		S		L		S		L		S		L		S		L		S		L		S		L		S		L		S		L		S	
	13	14	13	14	13	14	13	14	13	14	13	14	13	14	13	14	13	14	13	14	13	14	13	14	13	14	13	14	13	14	13	14	13	14	13	14
4777143	+	+	+	+	+	+	+	+	+	+	+	+	ns	+	+	+	ns	ns	ns	ns	+	+	+	+	+	+	+	+	+	+	+	+	+	+	+	+
100002778	+	+	+	+	+	+	+	+	+	+	+	+	ns	ns	+	+	ns	ns	ns	ns	+	+	+	+	+	+	+	+	+	+	+	+	+	+	+	+
2459933	+	+	+	+	+	+	+	+	+	+	+	+	ns	+	ns	+	ns	ns	ns	ns	+	+	+	+	+	+	+	+	+	+	+	+	+	+	ns	+
4590009	+	+	+	+	+	+	+	+	+	+	+	+	ns	+	+	+	ns	ns	ns	ns	ns	+	+	+	+	+	+	+	+	+	+	+	+	+	ns	+
4767650	+	+	+	+	+	+	+	+	+	+	+	+	ns	+	ns	ns	ns	ns	ns	ns	+	+	+	+	+	+	+	+	+	+	+	+	+	+	+	+
4777876	+	+	+	+	+	+	+	+	+	+	+	+	ns	+	ns	+	ns	ns	ns	ns	+	+	+	+	+	+	+	+	+	+	+	+	+	+	ns	+
4764736	+	+	+	+	+	+	+	+	+	+	+	+	ns	+	ns	+	ns	ns	ns	ns	+	+	+	+	+	+	+	+	+	+	+	+	+	+	ns	+
100002084	+	+	+	+	+	+	+	+	+	+	+	+	ns	+	ns	+	ns	ns	ns	ns	+	+	+	+	+	+	+	+	+	+	+	+	+	+	ns	+
4779820	+	+	+	+	+	+	+	+	+	+	+	+	ns	+	ns	+	ns	ns	ns	ns	+	+	+	+	+	+	+	+	+	+	+	+	+	+	ns	+
9671651	+	+	+	+	+	+	+	+	+	+	+	+	ns	+	ns	+	ns	ns	ns	ns	+	+	+	+	+	+	+	+	+	+	+	+	+	+	ns	+
100002221	+	+	+	+	+	+	+	+	+	+	+	+	ns	+	ns	+	ns	ns	ns	ns	+	+	+	+	+	+	+	+	+	+	+	+	+	+	ns	+
100002240	+	+	+	+	+	+	+	+	+	+	+	+	ns	+	ns	+	ns	ns	ns	ns	+	+	+	+	+	+	+	+	+	+	+	+	+	+	ns	+
100002307	+	+	+	+	+	+	+	+	+	+	+	+	ns	+	ns	+	ns	ns	ns	ns	+	+	+	+	+	+	+	+	+	+	+	+	+	+	ns	+
21693206	+	+	+	+	+	+	+	+	+	+	+	+	ns	+	ns	+	ns	ns	ns	ns	+	+	+	+	+	+	+	+	+	+	ns	+	+	+	+	+
9625858	+	+	+	+	+	+	+	+	+	+	+	+	ns	+	ns	ns	ns	ns	ns	ns	+	+	+	+	+	+	+	+	+	+	+	+	+	+	+	+
16723979	+	+	+	+	+	+	+	+	+	+	+	+	ns	ns	ns	+	+	+	+	+	+	+	+	+	+	+	+	+	ns	ns	ns	ns	+	+	+	+
4588115	+	+	+	+	+	+	+	+	+	+	+	+	ns	+	ns	+	ns	ns	ns	ns	+	+	+	+	+	+	+	+	+	+	+	+	+	+	ns	+
4578744	+	+	+	+	+	+	+	+	+	+	+	+	ns	+	ns	+	ns	ns	ns	ns	+	+	+	+	+	+	+	+	+	+	+	+	+	+	ns	+
9630313	+	+	+	+	+	+	+	+	+	+	+	+	ns	+	ns	+	ns	ns	ns	ns	+	+	+	+	+	+	+	+	+	+	+	+	+	+	ns	+
9685997	+	+	+	+	+	+	+	+	+	+	+	+	ns	+	ns	+	ns	ns	ns	ns	+	+	+	+	+	+	+	+	+	+	+	+	+	+	ns	+

7054160	+	+	+	+	+	+	+	+	+	+	+	+	ns	+	ns	+	ns	ns	ns	ns	+	+	+	+	+	+	+	+	+	+	+	+	+	+	ns	+
7047987	+	+	+	+	+	+	+	+	+	+	+	+	ns	+	ns	+	ns	ns	ns	ns	+	+	+	+	+	+	+	+	+	+	+	+	+	+	ns	+
4579908	+	+	+	+	+	+	+	+	+	+	+	+	ns	+	ns	+	ns	ns	ns	ns	+	+	+	+	+	+	+	+	+	+	+	+	+	+	ns	+
9676467	+	+	+	+	+	+	+	+	+	+	+	+	ns	+	ns	+	ns	ns	ns	ns	+	+	+	+	+	+	+	+	+	+	+	+	+	+	ns	+
2572101	+	+	+	+	+	+	+	+	+	+	+	+	ns	+	+	+	ns	ns	ns	ns	+	+	+	+	+	+	+	+	+	+	+	+	+	+	ns	ns
4772719	+	+	+	+	+	+	+	+	+	+	+	+	ns	+	ns	+	ns	ns	ns	ns	+	+	+	+	+	+	+	+	+	+	+	+	+	+	ns	+
4584050	+	+	+	+	+	+	+	+	+	+	+	+	ns	+	ns	+	ns	ns	ns	ns	+	+	+	+	+	+	+	+	+	+	+	+	+	+	ns	+
9693454	+	+	+	+	+	+	+	+	+	+	+	+	ns	+	ns	+	ns	ns	ns	ns	+	+	+	+	+	+	+	+	+	+	+	+	+	+	ns	+
4586378	+	+	+	+	+	+	+	+	+	+	+	+	ns	+	ns	+	ns	ns	ns	ns	+	+	+	+	+	+	+	+	+	+	+	+	+	+	ns	+
9718949	+	+	+	+	+	+	+	+	+	+	+	+	ns	+	ns	+	ns	ns	ns	ns	+	+	+	+	+	+	+	+	+	+	+	+	+	+	ns	+
2385022	+	+	+	+	+	+	+	+	+	+	+	+	ns	+	ns	+	ns	ns	ns	ns	+	+	+	+	+	+	+	+	+	+	+	+	+	+	ns	+
9713903	+	+	+	+	+	+	+	+	+	+	+	+	ns	+	ns	ns	ns	ns	ns	ns	+	+	+	+	+	+	+	+	+	+	+	+	+	+	+	+
100002927	+	+	+	+	+	+	+	+	+	+	+	+	ns	+	ns	+	ns	ns	ns	ns	+	+	+	+	+	+	+	+	+	+	+	+	+	+	ns	+
2532328	+	+	+	+	+	+	+	+	+	+	+	+	ns	+	ns	+	ns	ns	ns	ns	+	+	+	+	+	+	+	+	+	+	+	+	+	+	ns	+
100002943	+	+	+	+	+	+	+	+	+	+	+	+	ns	+	ns	+	ns	ns	ns	ns	+	+	+	+	+	+	+	+	+	+	+	+	+	+	ns	+
16721206	+	+	+	+	+	+	+	+	+	+	+	+	ns	+	ns	+	ns	ns	ns	ns	+	+	+	+	+	+	+	+	+	+	+	+	+	+	ns	+
100002966	+	+	+	+	+	+	+	+	+	+	+	+	ns	+	ns	+	ns	ns	ns	ns	+	+	+	+	+	+	+	+	+	+	+	+	+	+	ns	+
100002972	+	+	+	+	+	+	+	+	+	+	+	+	ns	+	ns	+	ns	ns	ns	ns	+	+	+	+	+	+	+	+	+	+	+	+	+	+	ns	+
100002973	+	+	+	+	+	+	+	+	+	+	+	+	ns	+	ns	+	ns	ns	ns	ns	+	+	+	+	+	+	+	+	+	+	+	+	+	+	ns	+
4774139	+	+	+	+	+	+	+	+	+	+	+	+	ns	+	ns	+	ns	ns	ns	ns	+	+	+	+	+	+	+	+	+	+	+	+	+	+	ns	+
100002999	+	+	+	+	+	+	+	+	+	+	+	+	ns	+	ns	ns	ns	ns	ns	ns	+	+	+	+	+	+	+	+	+	+	+	+	+	+	+	+
7059512	+	+	+	+	+	+	+	+	+	+	+	+	ns	+	ns	+	ns	ns	ns	ns	+	+	+	+	+	+	+	+	+	+	+	+	+	+	ns	+
9699244	+	+	+	+	+	+	+	+	+	+	+	+	ns	+	ns	+	ns	ns	ns	ns	+	+	+	+	+	+	+	+	+	+	+	+	+	+	ns	+
2512240	+	+	+	+	+	+	+	+	+	+	+	+	ns	+	ns	+	ns	ns	ns	ns	+	+	+	+	+	+	+	+	+	+	+	+	+	+	ns	+
9629163	+	+	+	+	+	+	+	+	+	+	+	+	ns	+	ns	+	ns	ns	ns	ns	+	+	+	+	+	+	+	+	+	+	+	+	+	+	ns	+
9624319	+	+	+	+	+	+	+	+	+	+	+	+	ns	+	ns	+	ns	ns	ns	ns	+	+	+	+	+	+	+	+	+	+	+	+	+	+	ns	+
2458442	+	+	+	+	+	+	+	+	+	+	+	+	ns	+	ns	+	ns	ns	ns	ns	+	+	+	+	+	+	+	+	+	+	+	+	+	+	ns	+
9667639	+	+	+	+	+	+	+	+	+	+	+	+	ns	+	ns	+	ns	ns	ns	ns	+	+	+	+	+	+	+	+	+	+	+	+	+	+	ns	+

9676081	+	+	+	+	+	+	+	+	+	+	+	+	ns	+	ns	+	ns	ns	ns	ns	+	+	+	+	+	+	+	+	+	+	+	+	+	+	ns	+
100011211	+	+	+	+	+	+	+	+	+	+	+	+	ns	+	ns	+	ns	ns	ns	ns	+	+	+	+	+	+	+	+	+	+	+	+	+	+	ns	+
100000002	+	+	+	+	+	+	+	+	+	+	+	+	ns	ns	ns	ns	ns	ns	ns	ns	+	+	+	+	+	+	+	+	+	+	+	+	+	+	+	+
7057018	+	+	+	+	+	+	+	+	+	+	+	+	ns	ns	ns	ns	ns	ns	ns	ns	+	+	+	+	+	+	+	+	+	+	+	+	+	+	+	+
100000140	+	+	+	+	+	+	+	+	+	+	+	+	ns	ns	ns	ns	ns	ns	ns	ns	+	+	+	+	+	+	+	+	+	+	+	+	+	+	+	+
4766144	+	+	+	+	+	+	+	+	+	+	+	+	+	+	+	+	ns	ns	ns	ns	ns	ns	ns	+	+	+	+	+	+	+	+	+	+	+	ns	+
2447999	+	+	+	+	+	+	+	+	+	+	+	+	ns	ns	ns	ns	ns	ns	ns	ns	+	+	+	+	+	+	+	+	+	+	+	+	+	+	+	+
4768594	+	+	+	+	+	+	+	+	+	+	+	+	ns	ns	ns	ns	ns	ns	ns	ns	+	+	+	+	+	+	+	+	+	+	+	+	+	+	+	+
9668081	+	+	+	+	+	+	+	+	+	+	+	+	ns	ns	ns	ns	ns	ns	ns	ns	+	+	+	+	+	+	+	+	+	+	+	+	+	+	+	+
2466550	+	+	+	+	+	+	+	+	+	+	+	+	ns	ns	ns	ns	ns	ns	ns	ns	+	+	+	+	+	+	+	+	+	+	+	+	+	+	+	+
4770353	+	+	+	+	+	+	+	+	+	+	+	+	ns	ns	ns	ns	ns	ns	ns	ns	+	+	+	+	+	+	+	+	+	+	+	+	+	+	+	+
4580300	+	+	+	+	+	+	+	+	+	+	+	+	ns	ns	ns	ns	ns	ns	ns	ns	+	+	+	+	+	+	+	+	+	+	+	+	+	+	+	+
2429206	+	+	+	+	+	+	+	+	+	+	+	+	ns	ns	ns	ns	ns	ns	ns	ns	+	+	+	+	+	+	+	+	+	+	+	+	+	+	+	+
7051388	+	+	+	+	+	+	+	+	+	+	+	+	ns	ns	ns	ns	ns	ns	ns	ns	+	+	+	+	+	+	+	+	+	+	+	+	+	+	+	+
2512042	+	+	+	+	+	+	+	+	+	+	+	+	ns	ns	ns	ns	ns	ns	ns	ns	+	+	+	+	+	+	+	+	+	+	+	+	+	+	+	+
9674157	+	+	+	+	+	+	+	+	+	+	+	+	ns	+	ns	+	ns	ns	ns	ns	ns	+	+	+	+	+	+	+	+	+	+	+	+	+	ns	+
7053864	+	+	+	+	+	+	+	+	+	+	+	+	ns	ns	ns	ns	ns	ns	ns	ns	+	+	+	+	+	+	+	+	+	+	+	+	+	+	+	+
9700949	+	+	+	+	+	+	+	+	+	+	+	+	ns	ns	ns	ns	ns	ns	ns	ns	+	+	+	+	+	+	+	+	+	+	+	+	+	+	+	+
4769898	+	+	+	+	+	+	+	+	+	+	+	+	ns	+	ns	ns	ns	ns	ns	ns	ns	+	+	+	+	+	+	+	+	+	+	+	+	+	+	+
16722541	+	+	+	+	+	+	+	+	+	+	+	+	ns	ns	ns	ns	ns	ns	ns	ns	+	+	+	+	+	+	+	+	+	+	+	+	+	+	+	+
7058229	+	+	+	+	+	+	+	+	+	+	+	+	ns	ns	ns	ns	ns	ns	ns	ns	+	+	+	+	+	+	+	+	+	+	+	+	+	+	+	+
100002333	+	+	+	+	+	+	+	+	+	+	+	+	ns	ns	ns	ns	ns	ns	ns	ns	+	+	+	+	+	+	+	+	+	+	+	+	+	+	+	+
100002803	+	+	+	+	+	+	+	+	+	+	+	+	ns	+	ns	+	ns	ns	ns	ns	ns	+	+	+	+	+	+	+	+	+	+	+	+	+	ns	+
100002833	+	+	+	+	+	+	+	+	+	+	+	+	ns	+	ns	+	ns	ns	ns	ns	ns	+	+	+	+	+	+	+	+	+	+	+	+	+	ns	+
100002891	+	+	+	+	+	+	+	+	+	+	+	+	ns	+	ns	+	ns	ns	ns	ns	ns	+	+	+	+	+	+	+	+	+	+	+	+	+	ns	+
2489041	+	+	+	+	+	+	+	+	+	+	+	+	ns	+	+	+	ns	ns	ns	ns	ns	+	ns	ns	+	+	+	+	+	+	+	+	+	+	+	+
9669763	+	+	+	+	+	+	+	+	+	+	+	+	ns	ns	ns	ns	ns	ns	ns	ns	+	+	+	+	+	+	+	+	+	+	+	+	+	+	+	+

2378069	+	+	+	+	+	+	+	+	+	+	+	+	ns	+	ns	+	ns	ns	ns	ns	ns	+	+	+	+	+	+	+	+	+	+	+	+	+	ns	+

[#] L - Łagiewniki, S - Smolice, [@] 13 - 2013, 14 - 2014, + - significant, ns - not-significant
